# Effects of Olive Oil on Markers of Inflammation and Endothelial Function—A Systematic Review and Meta-Analysis

**DOI:** 10.3390/nu7095356

**Published:** 2015-09-11

**Authors:** Lukas Schwingshackl, Marina Christoph, Georg Hoffmann

**Affiliations:** 1 Department of Nutritional Sciences, Faculty of Life Sciences, University of Vienna, Athanstraße 14 (UZAII), Vienna A-1090, Austria; E-Mails: lukas.schwingshackl@univie.ac.at (L.S.); a0902438@unet.univie.ac.at (M.C.); 2 Department of Epidemiology, German Institute of Human Nutrition Potsdam-Rehbruecke (DIfE), Arthur-Scheunert-Allee 114-116, 14558 Nuthetal, Germany

**Keywords:** cardiovascular disease, *C*-reactive protein, flow-mediated dilatation, interleukin-6, Mediterranean diet

## Abstract

The aim of the present systematic review was to synthesize data from randomized controlled trials investigating the effects of olive oil on markers of inflammation or endothelial function. Literature search in electronic databases Cochrane Trial Register, EMBASE, and MEDLINE was performed. Thirty studies enrolling 3106 participants fulfilled the selection criteria. Pooled effects of different interventions were assessed as mean difference using a random effects model. Olive oil interventions (with daily consumption ranging approximately between 1 mg and 50 mg) resulted in a significantly more pronounced decrease in *C*-reactive protein (mean difference: −0.64 mg/L, (95% confidence interval (CI) −0.96 to −0.31), *p* < 0.0001, *n* = 15 trials) and interleukin-6 (mean difference: −0.29 (95% CI −0.7 to −0.02), *p* < 0.04, *n* = 7 trials) as compared to controls, respectively. Values of flow-mediated dilatation (given as absolute percentage) were significantly more increased in individuals subjected to olive oil interventions (mean difference: 0.76% (95% CI 0.27 to 1.24), *p* < 0.002, *n* = 8 trials). These results provide evidence that olive oil might exert beneficial effects on endothelial function as well as markers of inflammation and endothelial function, thus representing a key ingredient contributing to the cardiovascular-protective effects of a Mediterranean diet. However, due to the heterogeneous study designs (e.g., olive oil given as a supplement or as part of dietary pattern, variations in control diets), a conservative interpretation of the results is necessary.

## 1. Introduction

Cardiovascular diseases (CVD) represent the main cause of disability and death in industrialized countries. Numerous risk factors are known to promote the pathogenesis of CVD. Some of these risk factors are modifiable lifestyle habits such as physical activity and choice of foods. Diets low in fruits, vegetables, nuts and seeds, whole grains, or *n*-3 rich seafood but high in sodium, salt, and sugar-sweetened beverages are regarded to increase the risk of CVD [1]. Thus, the lower incidence rates of CVD observed in Southern Europe might at least in part be explained by health-promoting dietary patterns such as the Mediterranean diet (MedD) rich in extra virgin olive oil, fruits, vegetables, nuts and seeds, legumes, but low in red meat and dairy products [2]. Extra virgin olive oil is the main source of fat in the MedD. With its high content in monounsaturated fatty acids and polyphenols, extra virgin olive oil might exert beneficial effects in the development and progression of diseases associated with chronic low-grade inflammation [3,4,5].

Low-grade, chronic synthesis and release of pro-inflammatory cytokines like interleukin-1 (IL-1) and tumor necrosis factor-α (TNF-α) within the vascular wall affect endothelial function e.g., via up-regulation of adhesion molecules [6]. Over time, this may lead to endothelial dysfunction, a predictor as well as a pathogenic mechanism of atherosclerosis and CVD [7]. Thus, excessive inflammatory processes of the endothelium were reported to be predictors of future cardiovascular events [8]. In a recent systematic review of randomized controlled studies (RCT) we showed that adherence to a MedD was associated with improvements in endothelial function as well as inflammatory status [9]. Beneficial effects of an MedD on markers of both inflammation and endothelial function have been reported by others as well [10,11,12]. One potential component mediating the positive effects of a MedD might be the prominent role of olive oil in this dietary pattern. Bioactive components of extra virgin olive oil have demonstrated endothelium-protective and anti-oxidative properties [4,13]. Therefore, consumption of extra virgin olive oil might explain the beneficial effects of a MedD with respect to incidences of CVD. To gain additional information about the effects of olive oil (a major component of the MedD) on parameters involved in the development of CVD, it was the aim of the present systematic review to synthesize data of randomized controlled trials investigating the effects of olive oil interventions (administered either in the form of capsules, supplemented to the habitual diet, or supplemented to a specific dietary pattern such as MedD) on markers of inflammation as well as endothelial function.

## 2. Experimental Section

This systematic review was registered in the PROSPERO International Prospective Register of Systematic Reviews [14], indexed under CRD42014008803.

### 2.1. Literature Search

Literature search was conducted using the electronic databases PubMed (1966 until June 2015), EMBASE (1980 until June 2015) and the Cochrane Trial Register (until June 2015). The following search terms were used: (“olive oil”) and (“endothelial” or “inflammation” or “CRP” or “*C*-reactive protein” or “FMD” or “flow-mediated dilatation”) and (“randomized controlled trial” or “randomized” or “clinical trials as topic” or “placebo” or “randomly” or “trial”) not (“animals”). Reference lists of retrieved articles, systematic reviews, and meta-analyses were checked for further relevant trials.

### 2.2. Study Selection

The following inclusion criteria were defined prior to study selection process:

(1) intervention with olive oil in pure form or as supplement (capsules); (2) randomized controlled trials (RCT) with either parallel or crossover design; (3) participants ≥19 years of age; (4) minimum intervention period of four weeks; (5) no other supplementation; (6) assessment of the “outcome of interest”: Markers of inflammation (*C*-reactive protein (CRP), interleukin-6 (IL-6), TNF-α, adiponectin) and endothelial function (intercellular adhesion molecule-1 (ICAM-1), vascular cell adhesion molecule-1 (VCAM-1), flow-mediated dilatation (FMD)); (7) report of post-intervention mean values (or if not available, change from baseline values were used instead) with standard deviation (or basic data which allow to calculate these parameters, *i.e.*, standard errors, 95% confidence interval, *p*-values).

### 2.3. Risk of Bias Assessment

The Cochrane Collaboration’s tool for assessing risk of bias was used to elucidate the risk of bias of the included studies attaching either low, unclear or high risk of bias to the seven domains (sequence generation, allocation concealment, blinding of participants and personnel, blinding of outcome assessment, incomplete outcome data, selective outcome reporting) to each study [15].

### 2.4. Data Extraction and Analyses

Data extracted from each trial were: first author’s family name; year of publication; number, age, and gender distribution of study participants; duration of intervention; description of olive oil intervention and respective control; outcomes and post-intervention mean values or differences in mean of two time points together with corresponding standard deviations.

All data were analyzed using the software REVIEW MANAGER 5.3. as provided by the Cochrane Collaboration [16]. In a random effects model, the post-mean values or the changes from baseline values and corresponding standard deviations of intervention and control/intervention groups were compared. Pooled effects of the different interventions were investigated as mean difference. Heterogeneity between trial results was tested with a standard χ^2^ test. The *I*^2^ parameter was used to quantify any inconsistency: *I*^2^ = ((Q − d*f*))/Q × 100%, where Q is the χ^2^ statistic and d*f* is its degrees of freedom. An *I*^2^-value of greater than 50% was considered to represent considerable heterogeneity.

Literature search was performed by Lukas Schwingshackl and Marina Christoph, while data extraction, analyses, and synthesis was done by all authors with disagreement resolved by consensus.

### 2.5. Specific Data Handling/Handling of Missing Data

After contacting the corresponding authors, the missing data of the trial by Damasceno *et al.* [17] as well as the CRP values of the trial by Sanders *et al.* [16] could be added to the meta-analysis.

## 3. Results

### 3.1. Literature Search and Study Characteristics

**Figure 1:**
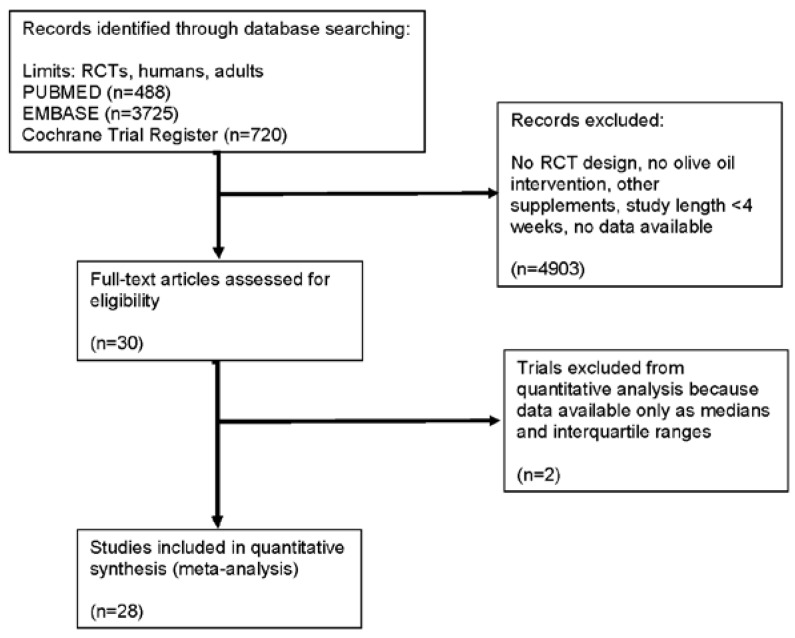
Flow diagram.

**Figure 2:**
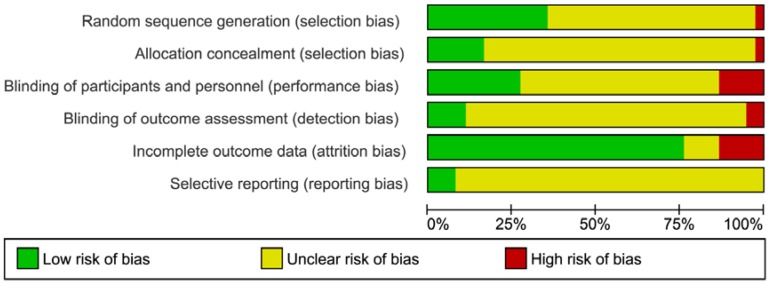

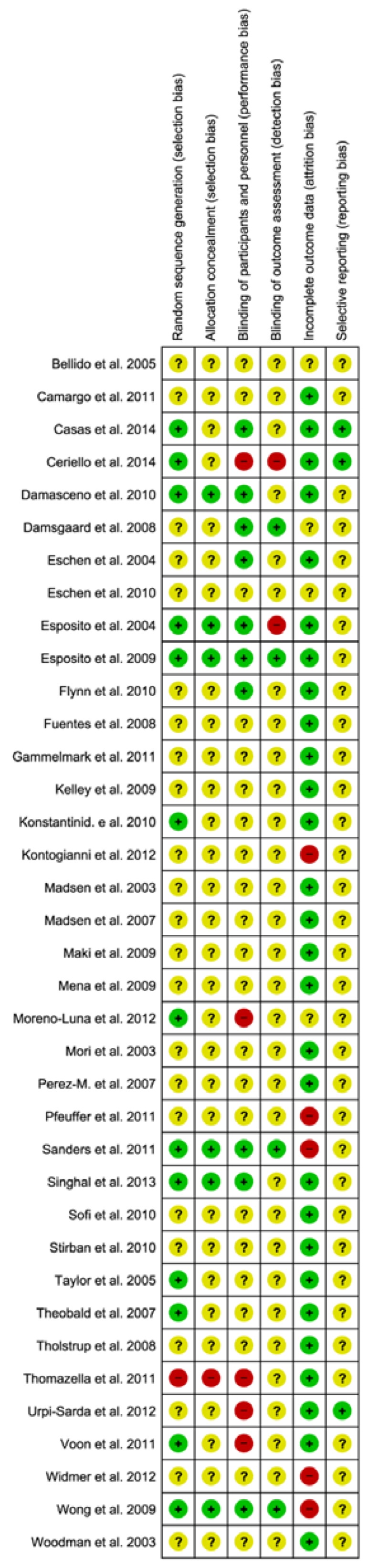
Risk of bias assessment tool. (**A)** Across trials, information is either from trials at low risk of bias (green), or from trials at unclear risk of bias (yellow), or from trials at high risk of bias (red). (**B)** For each study, every bias domain will be checked, the given summary represents an assessment of bias risk across studies. For each bias domain, low risk of bias means that information is from studies at low risk of bias (green), high risk of bias indicates the proportion of information from studies at high risk of bias which might be sufficient to affect the interpretation of the results (red), and unclear risk of bias refers to information from studies at low or unclear risk of bias (yellow).

Selection criteria were fulfilled by 30 studies with altogether 3106 participants [4,17,18,19,20,21,22,23,24,25,26,27,28,29,30,31,32,33,34,35,36,37,38,39,40,41,42,43,44,45,46]. The detailed steps of the selection process is summarized in Figure 1. Figure 2 summarizes the distribution of the risk of bias for all domains and all studies.

The study duration varied between four and 208 weeks. Part of the RCTs was comparing MedD supplemented with (virgin/extra virgin) olive oil and a control diet. Another subset of RCTs compared olive oil capsules against capsules containing other fats. Due to the different designs of the different interventions, the studies were classified in subgroups according to mode of olive oil intervention and controls as follows:

A Mediterranean diet (MedD) with a specified quantity of olive oil *vs.*

(1) low-fat diet;
(2) healthy diet;
(3) MedD and nuts;
(4) Western diet/diet high in carbohydrates;

A specified quantity of olive oil supplemented to the habitual diet *vs.*

(1) flaxseed oil;
(2) coconut oil/palm oil;
(3) olive oil with *n*-3 fatty acids;

A specified quantity of olive oil in capsules supplemented to the habitual diet *vs.*

(1) conjugated linoleic acid mix capsules;
(2) *n*-3 fatty acid capsules;

Or specific comparisons between plant-based diets with other dietary patterns, *i.e.*,

(1) plant-based olive oil diet *vs.* National Cancer Institute diet;
(2) monounsaturated fatty acid diet *vs.* saturated fatty acid diet and low-fat diet.

General study characteristics are summarized in Table 1.

**Table 1:** General characteristics of randomized controlled trials included in the systematic review.

Reference	Sample Size; % Female	Age (Years, Mean ± SD)	BMI (kg/m^2^, Mean ± SD)	Duration (Weeks), Design	Diseases/Health Status/Medication	Olive Oil Intervention	Comparison Group	Outcome Parameters
Casas *et al.*, 2014 [19]	164; olive oil: 56.4, control: 59.3	Olive oil: 68.1 ± 6, control: 67.4 ± 4	Olive oil: 27.9 ± 3.4, control: 26.5 ± 3.7	52, parallel	High risk for CVD (type 2 diabetes mellitus or 3 or more major cardiovascular risk factors)	MedD + extra virgin olive oil (50 mL/day)	Low-fat diet	CRP, sE-selectin, sP-selectin, sVCAM-1
Ceriello *et al.*, 2014 [20]	24; olive oil: 25, control: 33	n.d.	Olive oil: 29.8 ± 1.4, control: 29.2 ± 1.1	3 months, parallel	Type 2 diabetes mellitus	MedD + extra virgin olive oil (50 mL/day)	Low-fat diet	FMD
Damasceno *et al.*, 2010 [17]	18; 50	56 ± 13	25.7 ± 2.3	4, cross-over	Moderate hypercholesterolemia, no medication, no hormone replacement therapy	MedD + 30–50 g/day virgin olive oil (polyphenols 34.3 mg/100g)	MedD + walnuts (polyphenols 1.3 mg/100g); MedD + almonds (polyphenols 1.1 mg/100g)	CRP, sICAM-1, sVCAM-1
Damsgaard *et al.*, 2008 [21]	64; 0	Olive oil high linoleic acid: 24.9 ± 3.8, olive oil low linoleic acid: 25.5 ± 4.4, control high linoleic acid: 26.3 ± 4.8, control low linoleic acid: 24.9 ± 4.9	Olive oil high linoleic acid: 23.1 ± 1.9, olive oil low linoleic acid: 23.3. ± 1.9, control high linoleic acid: 21.9 ± 1.8, control low linoleic acid: 22.9 ± 1.9	8, 2 × 2 factorial	Healthy, no medication	5 mL/day unrefined extra virgin olive oil capsules	5 mL/day fish oil capsules	adiponectin, CRP, IL-6, VCAM-1
Damsgaard *et al.*, 2009 [22]	63; 0	Olive oil high linoleic acid: 24.9 ± 3.8, olive oil low linoleic acid: 25.5 ± 4.4, control high linoleic acid: 26.3 ± 4.8, control low linoleic acid: 24.9 ± 4.9	Olive oil high linoleic acid: 23.1 ± 1.9, olive oil low linoleic acid: 23.3. ± 1.9, control high linoleic acid: 21.9 ± 1.8, control low linoleic acid: 22.9 ± 1.9	8, 2 × 2 factorial	Healthy, no intensive sports	5 mL/day unrefined extra virgin olive oil capsules	5 mL/day fish oil capsules	
Eschen *et al.*, 2004 [23]	60; olive oil: 82, control: 66	Olive oil: 39 ± 10, control: 38 ± 11	Olive oil: 24.6 ± 3.7, control:25.1 ± 2.9	12, parallel	Healthy, no medication	10 g/day olive oil capsules	6.6 g/day *n*-3 PUFA capsules; 2.0 g/day *n*-3 PUFA + 7 g/day olive oil capsules	ICAM-1, VCAM-1, P-selectin
Eschen *et al.*, 2010 [24]	138; olive oil: 22, control: 13	Olive oil: 61 ± 8, control: 58 ± 10	Olive oil: 27.1 ± 0.4, control: 27.1 ± 0.5	24, parallel	Chronic heart failure and left ventricular ejection fraction<40 or stable oral medication	1 g/day olive oil capsules	0.9 g/day *n*-3 PUFA capsules	SICAM-1, sVCAM-1, sP-selectin
Esposito *et al.*, 2004 [25]	180; olive oil: 46, control: 44	Olive oil: 44.3 ± 6.4, control: 43.5 ± 5.9	Olive oil: 27.9 ± 3.4, control: 28.1 ± 3.2	104, parallel	≥3 criteria of metabolic syndrome, no medication, no intensive sports	MedD + olive oil	Generally healthy diet	hsCRP, IL-6
Esposito *et al.*, 2009 [26]	195; olive oil: 50, control: 51.5	n.d.	n.d.	208, parallel	Overweight, newly diagnosed type 2 diabetes mellitus, no medication	MedD + 30–50 g olive oil/day	Low-fat diet	adiponectin
Flynn *et al.*, 2010 [27]	28; n.d.	59.2 ± 6.1	27.9 ± 2.8	8 weight loss, 24 follow-up, cross-over	Overweight women with invasive breast cancer, stable medication	Plant-based olive oil diet with at least 3 tablespoons extra virgin olive oil/day	National Cancer Institute diet with canola oil	CRP
Fuentes *et al.*, 2008 [28]	20; n.d.	23.3. ± 1.5	24.65 ± 2.91	4, cross-over	Healthy, no medication, no intensive sports	MUFA diet based on extra virgin olive oil (38% fat)	SFA diet; low-fat *n*-3 enriched diet based on α-linoleic acid	sVCAM-1, sICAM-1
Gammelmark *et al.*, 2012 [29]	50; olive oil: 52, control: 52	Olive oil: 58.0 ± 17.4, control: 55.4 ± 9.5	Olive oil: 30.8 ± 4.2, control: 29.5 ± 3.3	6, parallel	Abdominally obese, women postmenopausal, no medication except NSAIDs	2 g/day olive oil capsules	2 g/day fish oil capsules	Adiponectin, CRP, IL-6
Konstantinidou *et al.*, 2010 [30]	90; olive oil: 67, control: 63	Olive oil: 45 ± 10, control: 44 ± 10	Olive oil: 25 ± 4, control: 26 ± 5	12, parallel	Healthy	MedD + virgin olive oil (phenolic content 328 mg/kg)	MedD + washed virgin olive oil (phenolic content 55 mg/kg); habitual diet	sP-selectin
Kontogianni *et al.*, 2013 [31]	37; 78	25.6 ± 5.9	21.9 ± 2.5	6, cross-over	Healthy, no medication, no intensive sports	15 mL/day extra virgin olive oil	15 mL/day flaxseed oil	Adiponectin, CRP, TNF-α
Maki *et al.*, 2009 [32]	76; olive oil: 80, control: 88	Olive oil: 49.6 ± 1.4, control: 49.4 ± 1.7	Olive oil: 31.1 ± 1.1, control: 32.6 ± 1.5	4, parallel	Healthy, abdominally obese	2 g/day olive oil capsules	2 g/day krill oil capsules; 2 g/day menhaden oil capsules	CRP
Mena *et al.*, 2009 [33]	106; olive oil: 84, control: 76	Olive oil: 66 ± 11, control: 66 ± 7	Olive oil: 28.0 ± 2.9, control: 27.8 ± 3.2	12, parallel	Type 2 diabetes mellitus or ≥3 CVD risk factors, ACE inhibitors, diuretics, anti-hypertensive agents, statins, lipid-lowering agents, insulin, oral glucose-lowering agents, aspirin or anti-platelet drugs	MedD + virgin olive oil (1 L/week)	MedD + nuts (30 g/day); low-fat diet	sE-selectin, sP-selectin, sVCAM-1, sICAM-1, IL-6, CRP
Mori *et al.*, 2003 [34]	51; olive oil: 25, control: 28	Olive oil: 61.5 ± 1.9, control: 60.9 ± 1.9	Olive oil: 29.9 ± 1.0, control: 28.6 ± 0.7	6, parallel	Hypertension, type 2 diabetes mellitus, anti-hypertensive therapy, oral glucose-lowering agents but no insulin	4 g/day olive oil capsules	4 g/day EPA capsules; 4 g/day DHA capsules	CRP, IL-6, TNF-α
Perez-Martinez *et al.*, 2007 [2]	16; 0	n.d.	n.d.	4, cross-over	Healthy, no medication, no intensive sports	MedD + virgin olive oil	SFA diet; diet high in carbohydrates	VCAM-1
Pfeuffer *et al.*, 2011 [35]	85; olive oil: 0, control: 0	n.d.	Conjugated linoleic acid: 28.3 ± 2.3, safflower oil: 28.2 ± 2.0, heated safflower oil: 28.9 ± 2.6	4, parallel	Overweight or obese, metabolic syndrome, coronary heart disease	4.5 g/day olive oil capsules	4.5 g/day conjugated linoleic acid mixture capsules; 4.5 g/day safflower oil capsules; 4.5 g/day heated safflower oil capsules	CRP, sICAM, sVCAM, sE-selectin
Sanders *et al.*, 2011 [18]	367; olive oil: 61, control: 56	Olive oil: 55 (range: 54–57), control: 55 (range: 54–57)	Olive oil, males: 27 (range: 26–28), olive oil, females: 26 (range: 25–27), control, males: 26 (range: 25–27), control, females: 25 (range: 24–26)	52, parallel	No CVD, medication: statins, anti-hypertensive medication, hormone replacement therapy, thyroxine	3 g/day refined olive oil capsules	0.45/0.9/1.8 g/day EPA + DHA capsules	CRP, FMD
Singhal *et al.*, 2013 [36]	324; olive oil: 60, control: 66	Olive oil: 27.6 ± 4.7, control: 28.2 ± 4.8	Olive oil: 23.6 ± 3.5, control: 23.6 ± 34.3	16, parallel	Healthy, no medication	4 g/day olive oil capsules	1.6 g/day DHA + 2.4 g/day carrier oil capsules	CRP, FMD
Sofi *et al.*, 2010 [37]	11; olive oil: 0, control: 33	Olive oil: 54 (range: 42–70), control: 55 (range: 30–41)	Olive oil: 29.3 ± 3.9, control: 29.3 ± 4.1	52, parallel	Non-alcoholic fatty liver disease	6.5 mL/day olive oil	6.5 mL/day olive oil enriched with *n*-3 PUFA	adiponectin
Stirban *et al.*, 2010 [38]	34; n.d.	56.8 ± 8.3	31.3 ± 4.1	6, cross-over	Type 2 diabetes mellitus, aspirin, angiotensin-converting enzyme inhibitors, angiotensin receptor blockers, calcium channel blockers, ß-blockers, diuretics, statins	2 g/day olive oil capsules	2 g/day *n*-3 PUFA capsules	FMD
Taylor *et al.*, 2005 [39]	40; olive oil: 0, control: 0	Olive oil: 47 ± 8, control: 45 ± 6	Olive oil: 33 ± 3, control: 33 ± 3	12, parallel	Overweight, healthy	4.5 g/day olive oil capsules	4.5 g/day conjugated linoleic acid mixture capsules	adiponectin, CRP, FMD, TNF-α
Theobald *et al.*, 2007 [40]	39; 50	Male: 51.1 ± 7.4, female: 46.2 ± 4.9	Male: 51.1 ± 7.4, females: 46.2 ± 4.9	12, cross-over	Healthy, no medication	1.5 g/day refined olive oil capsules	0.7 g/day DHA capsules	IL-6, CRP, sE-Selectin
Tholstrup *et al.*, 2008 [41]	69; olive oil: 100, control: 100	Olive oil: 59.9 ± 4.9, control: 62.3 ± 5.0	Olive oil: 25.5 ± 3.3, control: 25.6 ± 3.1	16, parallel	Healthy, postmenopausal women, no medication	5.5 g/day olive oil capsules	5.5 g/day conjugated linoleic acid mixture capsules; 5.5 g/day conjugated linoleic acid milk capsules	IL-6, ICAM-1, VCAM-1
Thomazella *et al.*, 2011 [42]	40; olive oil: 0, control: 0	Olive oil: 55.0 ± 4.6, control: 54.6 ± 5.0	Olive oil: 26.5 ± 1.9, control: 26.3 ± 2.5	12, parallel	≥1 coronary event, clinical stablility, aspirin, anti-platelet drugs, statins (+ezetimibe), nitrates, ACE inhibitors, *ß* blockers	MedD + extra virgin olive oil (30 mL/day)	Low-fat therapeutic lifestyle changes diet	CRP, sICAM-1, sVCAM-1, FMD
Urpi-Sarda *et al.*, 2012 [43]	516; n.d.	n.d.	n.d.	52, parallel	Type 2 diabetes mellitus or ≥3 CVD risk factors	MedD + virgin olive oil (1 L/week)	MedD + nuts; low-fat diet	ICAM-1, IL-6
Voon *et al.*, 2011 [44]	45; 80	30.1 ± 8.3	30.1 ± 8.3	5, cross-over	healthy	Olive oil (2/3 of total fat)	Coconut oil (2/3 of total fat); palm olein (2/3 of total fat)	CRP, IL-6, TNF-α
Wong *et al.*, 2010 [45]	97; olive oil: 58, control: 53	Olive oil: 59.0 ± 8.3, control: 61.2 ± 9.0	Olive oil: 26.4 ± 4.4, control: 25.2 ± 3.7	12, parallel	Type 2 diabetes mellitus, no cardiovascular events, oral glucose-lowering agents/insulin	4 g/day olive oil capsules	4 g/day fish oil capsules	CRP, FMD
Woodman *et al.*, 2003 [46]	59; olive oil: 25, control: 28	Olive oil: 61.5 ± 7.6, control: 60.9 ± 8.2	Olive oil: 29.9 ± 4.0, control: 30.6 ± 3.1	6, parallel	Obese, type 2 diabetes mellitus and hypertension, oral glucose-lowering agents and treatment for hypertension	4 g/day olive oil capsules	4 g/day EPA capsules; 4 g/day DHA capsules	FMD, P-selectin

ACE = angiotensin converitng enzyme; CRP= C-reactive protein; CVD = cardiovascular disease; DHA = docosahexaenoic acid; EPA = eicosapentaenoic acid; FMD = flow-mediated dilatation; ICAM-1 = intercellular adhesion molecule-1; IL-6 = interleukin-6; MedD = Mediterranean diet; NSAIDs = non-steroidal anti-inflammatory drugs; PUFA = polyunsaturated fatty acids; TNF-α = tumor necrosis factorα; VCAM-1 = vascular cell adhesion molecule-1.

### 3.2. Markers of Inflammation

**Figure 3:**
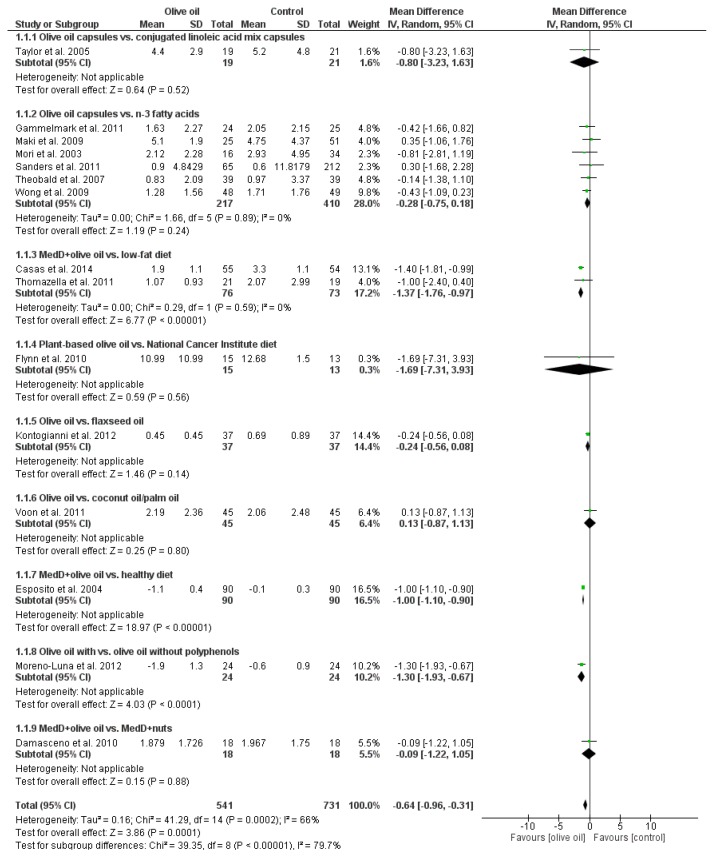
Effects of olive oil on C-reactive protein (mg/L). Forest plot showing pooled mean differences with 95% confidence intervals (CI) for 14 randomized controlled diets. For each study, the shaded square represents the point estimate of the intervention effect. The horizontal line joins the lower and upper limits of the 95% CI of these effects. The area of the shaded square reflects the relative weight of the study in the respective meta-analysis. The diamond at the bottom of the graph represents the pooled MD with the 95% CI for all study groups. MedD = Mediterranean diet.

**Figure 4:**
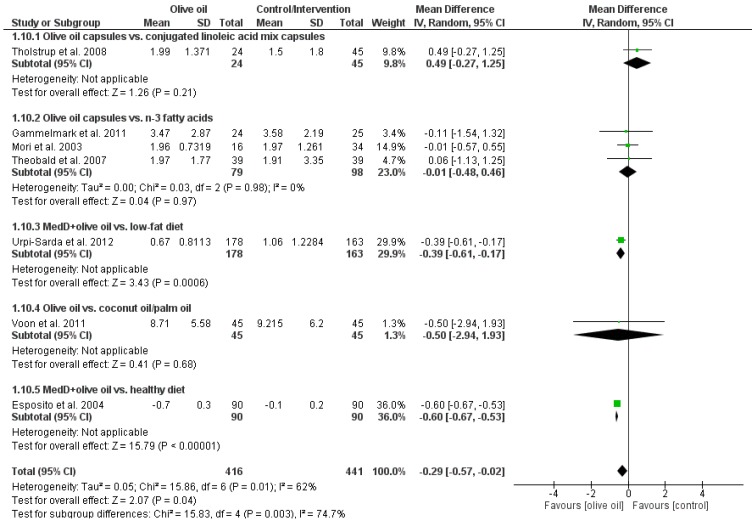
Effects of olive oil on interleukin-6 (pg/mL). Forest plot showing pooled mean differences with 95% confidence intervals (CI) for seven randomized controlled diets. For each study, the shaded square represents the point estimate of the intervention effect. The horizontal line joins the lower and upper limits of the 95% CI of these effects. The area of the shaded square reflects the relative weight of the study in the respective meta-analysis. The diamond at the bottom of the graph represents the pooled MD with the 95% CI for all study groups. MedD = Mediterranean diet.

Olive oil interventions resulted in a significantly more pronounced decrease in CRP (MD: −0.64 mg/L, (95% CI −0.96 to −0.31), *p* < 0.0001, *I*^2^ = 66%) as compared to the respective controls (Figure 3). Likewise, decreases in IL-6 levels were significantly stronger in the olive oil intervention groups as compared to controls (MD: −0.29 (95% CI −0.7 to −0.02), *p* < 0.04, *I*^2^ = 62%) (Figure 4). For both outcome parameters, the largest number of studies used capsules as a mode of application and compared olive oil with *n*-3 fatty acids. Regarding this subgroup, mean differences were (−0.28 (95% CI −0.75 to 0.18), *p* < 0.24, *I*^2^ = 0%) for CRP and (−0.01 (95% CI −0.48 to 0.49), *p* < 0.97, *I*^2^ = 0%) for IL-6, respectively. Pooled estimates of effects size for all markers of inflammation are summarized in Table 2.

**Figure 5:**
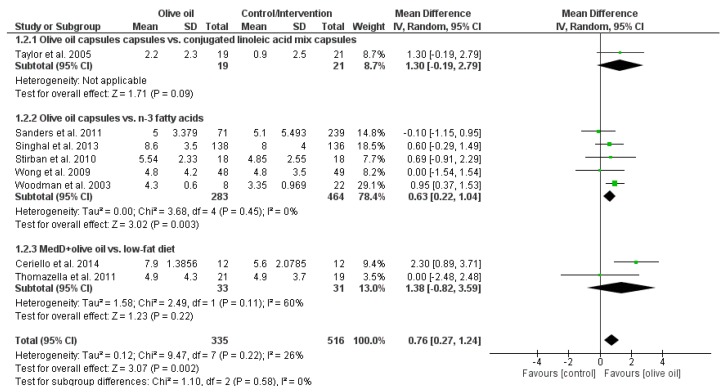
Effects of olive oil on flow-mediated dilatation (%, absolute percentage). Forest plot showing pooled mean differences with 95% confidence intervals (CI) for eight randomized controlled diets. For each study, the shaded square represents the point estimate of the intervention effect. The horizontal line joins the lower and upper limits of the 95% CI of these effects. The area of the shaded square reflects the relative weight of the study in the respective meta-analysis. The diamond at the bottom of the graph represents the pooled MD with the 95% CI for all study groups. Please note that the labeling of the X-axis has been switched as compared to Figure 3 and Figure 4, respectively, since for flow-mediated dilatation an increase is considered to be favorable. MedD = Mediterranean diet.

FMD values (MD: 0.76 (95% CI 0.27 to 1.24), *p* < 0.002, *I*^2^ = 26%) were more increased in individuals subjected to olive oil interventions (Figure 5). With respect to subgroups comparing olive oil with *n*-3, changes in FMD were significantly more pronounced in the olive oil groups when compared to their respective controls as well (MD: 0.63 (95% CI 0.22 to 1.04), *p* < 0.003, *I*^2^ = 0%). Results for other markers of endothelial function are given in Table 2.

**Table 2:** Pooled estimates of effect size for the results of olive oil interventions compared to respective controls.

Outcome Parameter	Mean Difference	95% Confidence Interval	*p*-Value	No. of Studies	Sample Size	*I*^2^ (%)
C-reactive protein (mg/L)	−0.64	(−0.96, −0.31)	0.0001	15	1272	66
Adiponectin (mg/L)	0.44	(−0.20, 1.09)	0.18	6	313	56
Interleukin-6 (pg/mL)	−0.29	(−0.57, −0.02)	0.04	7	857	62
Tumor necrosis factor-α (µg/L)	0.02	(−0.02, 0.07)	0.36	5	303	95
sE-Selectin (ng/mL)	−3.16	(−4.07, −2.25)	0.00001	2	187	0
sP-Selectin (ng/mL)	10.78	(4.01, 17.54)	0.002	4	358	41
sICAM-1 (ng/L)	−0.02	(−0.04, 0.00)	0.02	7	724	84
sVCAM-1 (ng/L)	−0.02	(−0.05, 0.01)	0.14	8	524	37
FMD (%)	0.76	(0.27, 1.24)	0.002	8	851	26

FMD = flow-mediated dilatation; *I*^2^ = inconsistency (heterogeneity); ICAM-1 = Intercellular adhesion molecule-1; VCAM-1 = Vascular cell adhesion molecule-1.Markers of endothelial function.

### 3.3. Publication Bias

**Figure 6:**
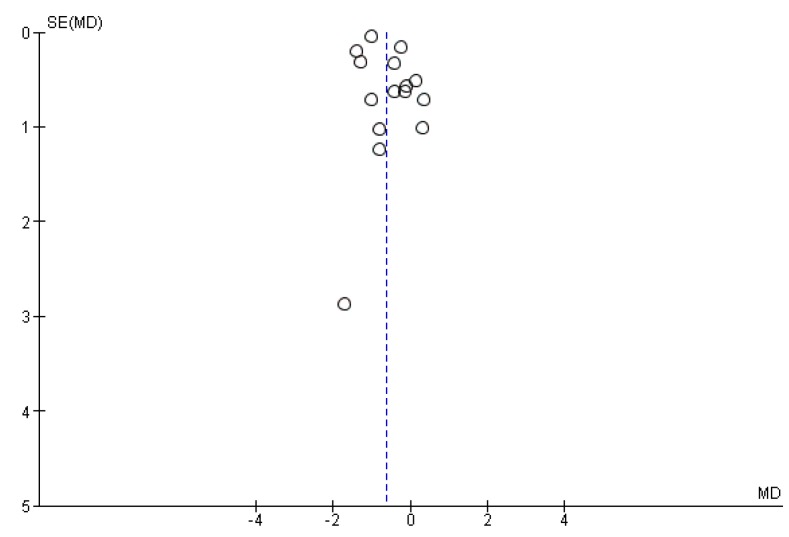
Funnel plot showing study precision against the mean differences effect estimate with 95% confidence intervals for *C*-reactive protein. SE = Standard error.

A funnel plot was generated for CRP (Figure 6), the only parameter assessed in at least 10 different trials. The plot indicates moderate asymmetry, thus a publication bias such as the difficulty in publishing negative or inconclusive data cannot be excluded to have an effect on the present meta-analyses.

## 4. Discussion

Synthesis of data available from RCTs in the present systematic review suggest that markers of inflammation (CRP, IL-6) and those characterizing endothelial function (FMD, sE-Selectin) were favorably affected following interventions with olive oil. Although it is likely that no single biomarker is able to represent all the important risk information, most of the outcome parameters taken into consideration in this meta-analysis are regarded to be valid indicators of inflammation and endothelial dysfunction.

The association between serum CRP concentrations and cardiovascular risk has been suggested by various studies, and elevated CRP levels are regarded to be an independent risk factor for CVD [47,48]. The US Preventive Services Task Force performed a meta-analysis of 22 studies showing that CRP concentrations greater than 3.0 mg/L were associated with an approximate 60% excess risk of incident coronary heart disease as compared to levels less than 1 mg/L [49]. In addition, serum CRP levels can predict long-term risk of incidence of myocardial infarction, ischemic stroke, peripheral vascular disease and all-cause mortality [50]. Although the value of CRP as a predictor for CVD is still discussed controversially, decreases in CRP values found in the present meta-analysis may support the concept of a cardio-protective effect of olive oil intake. In addition to its function as a stimulator of CRP synthesis [51,52], IL-6 has been shown to correlate with an increased risk of coronary heart disease in prospective studies [53]. Moreover, increased baseline levels of IL-6 were found to predict future cardiovascular events [54].

The other inflammatory markers investigated in this systematic review were unaffected by olive oil interventions. Although TNF-α is a relevant trigger during the inflammatory response, it has only rarely been assessed in epidemiological studies [55]. Likewise, data on a potential association between adiponectin and CVD risk are inconclusive [56,57,58]. An independent association of hypoadiponectinaemia with endothelial dysfunction measured by FMD has been observed by Tan *et al.* [59] in diabetic patients. Changes in TNF-α and adiponectin did not differ between olive oil interventions and respective controls in the present meta-analyses, which might be explained by the low number of study participants enrolled in the RCTs assessing these parameters.

To assess endothelial function, one of the standard non-invasive tools is FMD, which is regarded to reflect the local bioavailability of endothelium-derived vasoactive substances such as nitric oxide or endothelin-1. Reduced values of FMD are regarded to be early markers of atherosclerosis [60] as well as a predictor of future CVD events [8,61]. The association between reduced FMD and cardiovascular risk in individuals with varying baseline risk was demonstrated by several studies [62,63,64]. In a meta-analysis by Inaba and co-workers [8] synthesizing data of 5,500 participants of observational studies, each 1% reduction of FMD was associated with a 13% risk increase for cardiovascular events. This would equal an approximately 10% risk reduction given the effects of olive oil on FMD in the present meta-analysis.

Selectins are primary adhesion molecules in the inflammatory process expressed on the surfaces of activated endothelial cells, platelets, and leukocytes upon stimulation by TNF-α, IL-6, and other pro-inflammatory cytokines [65]. Elevated concentrations of E-selectin were found to be associated with ischemic events independent of traditional risk markers in the PRIME study [66,67]. ICAM-1 and VCAM-1 promote the adhesion of leukocytes to the endothelium. They are both up-regulated by pro-inflammatory cytokines, although VCAM-1 is considered to be expressed in more advanced states of atherosclerosis. This might explain at least in part why, in contrast to ICAM-1, reductions in VCAM-1 levels were not significantly more pronounced following olive oil interventions in the present systematic review.

Vascular reactivity is affected by food intake. Atherosclerotic events may be slowed down by anti-oxidant compounds in food via limiting oxidative damage and restoring endothelial function [68]. Thus, polyphenol intake has been associated with low mortality rates caused by coronary heart disease [69]. Other studies indicate that endothelial function and lipid profile were improved by anti-oxidant and anti-inflammatory polyphenols [70]. Therefore, the phenolic compounds present in extra virgin olive oil might mediated the beneficial effects observed in the present meta-analyses. Extra virgin olive oil polyphenols demonstrated strong anti-oxidant properties in experimental studies [3,13]. *In vivo* studies in healthy volunteers and patients with hypercholesterolemia or stable coronary heart disease have demonstrated that polyphenols improve ischemic reactive hyperemia blood pressure as well as inflammatory status [4,71].

Another potential health-promoting ingredient of olive oil is oleic acid. High oleic acid content of olive oil was demonstrated to affect metabolic functions and cardiovascular risk factors [72]. In various meta-analyses and meta-regressions, beneficial effects of monounsaturated fatty acids such as oleic acid on cardiovascular risk factors have been reported although the data available at present are still ambiguous [5,9,73].

### Limitations of the Systematic Review

Although randomized controlled studies are regarded to prove a high level of evidence, the present systematic review has some major limitations as well. Various outcome parameters were associated with I^2^ values higher than 50%, a threshold predetermined to indicate a considerable amount of heterogeneity. Trials varied with respect to study design, e.g., length of intervention, amount and type of olive oil used, classification of alternate source of fat (“control”), number of participants. Moreover, some study designs prescribed the intake of (extra virgin) olive oil provided by the investigators in pre-defined amounts (e.g., 30–50 g/day, 50 mL/day, or 1 L/week) to ensure a regular intake in combination with a MedD. However, besides vegetables, fruits, plant proteins, fish or whole grains, the traditional MedD is usually characterized by a high intake of olive oil in itself. Thus, these trials most likely have two separate sources of olive oil, which makes it impossible to quantify the absolute intake. Unfortunately, heterogeneous study protocols are a common characteristic in nutritional interventions. In addition, most studies failed to provide enough information for a thorough risk of bias analysis (Figure 1) and publication bias could only be assessed for one measured quantity, *i.e.*, CRP. Some outcome parameters were collected in a rather low number of studies with a restricted sample size. This may at least in part explain another limitation of our study, *i.e.* the difficulty to model a link between biomarkers of inflammation and those of endothelial function via synthesizing the corresponding data from trials with heterogeneous designs. Taken together, these limitations implicate a cautious interpretation of the results of our meta-analyses.

## 5. Conclusions

The present systematic reviews provides some evidence that olive oil might exert beneficial effects on markers of inflammation and endothelial function. Since improvements in these parameters have been described in individuals adhering to a Mediterranean diet, olive oil might represent a key ingredient of this dietary pattern mediating these favorable effects.
